# Mental hospital reform in Asia: the case of Yuli Veterans Hospital, Taiwan

**DOI:** 10.1186/1752-4458-3-1

**Published:** 2009-01-02

**Authors:** Chih-Yuan Lin, Ai-Ling Huang, Harry Minas, Alex Cohen

**Affiliations:** 1 Yuli Mental Health Research Center, Yuli Veterans Hospital, Hualien, Taiwan. Graduate Institute of Humanities in Medicine, Taipei Medical University, Taiwan; 2 Centre for International Mental Health, Melbourne School of Population Health, The University of Melbourne, Carlton Vic 3010, Australia; 3 Nutrition and Public Health Intervention Research Unit, London School of Hygiene & Tropical Medicine, London WC1E 7HT, UK

## Abstract

**Background:**

Yuli Veterans Hospital (YVH) has been the largest mental hospital for the patients with chronic and severe mental illness in Taiwan for the past 50 years. While this hospital used to be a symbol of hopelessness among patients and their families and an unspoken shame among Taiwan psychiatry and mental health circles it now represents an example of how an old, custodial hospital can be transformed into a very different institution. In this case study we will describe the features of this transformation, which, over the past 20 years, has aimed to help extended stay inpatients with severe mental illness to integrate into the local community of Yuli even though it is not their original home.

**Methods:**

Using historical documents and oral narratives from Yuli inhabitants, workers and patients of YVH, we will offer a case study of the Yuli model.

**Results:**

There are four main components of the Yuli model: holistic medical support, vocational rehabilitation, case management, and the residential program. The four components help patients recover two essential features of their lives: vocational life and ordinary daily routines. As the process of recovery evolves, patients gradually regain inner stability, dignity, self-confidence, and a sense of control. The four components are critical to rebuild the structure and order of life of the patients and are indispensable and interdependent parts of one service package. They operate simultaneously to benefit the patients to the greatest degree possible.

**Discussion:**

There are many challenges to the further development and financial viability of the model of services developed at YVH. There are also important questions concerning the replicability of the Yuli model in other sociocultural and service system contexts.

**Conclusion:**

This case study reveals the possibility of transforming a custodial mental hospital into a hospital providing high quality care. Hospital and community are not in opposition. They are part of a continuum of care for the patients. We reinterpret and redefine the boundary and function of hospital and community, and thereby create a new service model, the Yuli Model, to help patients to reintegrate into the community. The Yuli model, which particularly focuses on the needs of people with long-standing illness and prolonged hospital stay, illustrates one approach to linking hospital and community in a creative and constructive manner.

## Background

For at least 200 years, psychiatry has grappled with the challenge of reforming the institutions devoted to the care of mentally ill persons. Beginning in the late 18th Century – with the work of Chiarugi in Italy [1], Pinel in France [2], and Tuke in England [3] – there were efforts throughout North America and Europe to abolish the brutal conditions in "madhouses" and hospitals for the insane, and to introduce humane forms of treatment and care [4]. Beginning in the early 19th Century, calls began in England and France to develop systems of state-run asylums which would, presumably, be free of the abuses in private, non-regulated facilities [4,5]. These asylums were originally planned to be fairly small (in the range of 200–250 beds) but a variety of pressures – economic, political, professional, as well as demand for services – resulted in overcrowding and rapid expansion in the size of mental hospitals [6,7]. Deteriorating conditions were coupled, by the 1860's and 1870's, with a growing pessimism about whether mental hospitals provided treatments that resulted in high rates of recovery. In the United States, this shift from optimism to pessimism was personified by Pliny Earle who was an early champion of the curative powers of the hospital [8], but who later revealed, through a careful study of hospital statistics, that rates of cure were woefully low [9]. In the words of Gerald Grob, "Slowly the positive images of hospitals that had prevailed in the mid-nineteenth century gave way to far more negative ones associated with hopelessness, abuse, and ultimately death" [6]. Another historian of psychiatry, Andrew Scull, commented more harshly about this transition: "the rapid collapse of the asylum's pretensions to provide cure in the post-1845 era had been matched by the decay and disappearance of all the crucial features of moral treatment – those elements that were supposed to distinguish the asylum from the prison" [4].

The existence of the mental hospital was not confined to North America and Europe. Along with the advance of colonial power into Asia went the practice of psychiatry. The British established mental hospitals in India [10], Singapore [11], and Hong Kong [12], and the Dutch did the same in Indonesia [13]. The situation in China was slightly different – the original impetus for hospitals came from Western religious missionaries – but ultimately had the same result [14]. At the present time, the care and treatment offered in these hospitals do not, in general, conform with the recommendations of the WHO's *World Health Report 2001 *[15] to downsize mental hospitals, to shift patient care into the community, and to depend on general hospitals for treatment of acute psychiatric conditions. At worst, and as documented in reports by the National Human Rights Commission of India [16] and in TimeAsia [17,18], the care offered by, and the conditions in the large hospitals of Asia are, too often, less than ideal and marked by human rights violations. Therefore, improving conditions in the psychiatric hospitals of Asia and moving toward mental health systems that provide high quality care, must be considered one of the great challenges facing Asian psychiatry.

An example of how that reform can be accomplished may be found at Yuli Veterans Hospital (YVH), which is located at the midpoint of Taiwan's East Rift Valley in the town of Yuli, about one and half hours by road south of Hualien and about the same distance north of Taitung. With barely 30,000 inhabitants, the town of Yuli has been home to multiple groups of people, such as the Fukienese and Hakka who have emigrated from Mainland China, and various aboriginal tribes. In addition, thousands of people who suffer from chronic and severe mental illness have been transferred from institutions throughout Taiwan to Yuli. The hospital represents a remarkable example of how a custodial hospital can be transformed into a very different institution.

## Methods

In this case study we will describe the features and development of the Yuli model, whose goal is to help extended stay inpatients with severe mental illness integrate into the local community of Yuli even though it is not their original home. Using historical documents and oral narratives from Yuli inhabitants, workers and patients of YVH, we will offer a detailed picture of this model.

## Results

### The historical context of the Yuli model

YVH was established in 1957. At first, the patients were veteran soldiers who had retreated from Mainland China between 1948 and 1949. The hospital workers were also veterans; they and their families lived around the main campus of hospital. Thus, the patients and personnel of the hospital were recent immigrants to Taiwan, in contrast to the original inhabitants of Yuli. According to former director of the Division of Rehabilitation, Mr. Zou, Ru-Zhe, himself a veteran from Mainland China who came to Yuli five months after the hospital opened, YVH was designated from the very beginning as the only veterans hospital devoted to the care and treatment of mental illnesses. Initially, the government had tried to establish a psychiatric hospital in the western counties but, when it encountered strong opposition to the plan, chose Yuli, which was remote, and had a small population that did not mount serious opposition to the proposed hospital. When Mr. Zou first arrived, the hospital already had more than 1,100 patients. This number kept increasing until it reached its peak of 4,060 in the 1960s.

In 1958, it took Mr. Zou almost one day to travel from Taipei to Yuli. When he arrived at the hospital he was scared by its appearance. Police were stationed at the main gate to the campus, giving the impression that this was a prison not a hospital. It was only when he saw patients singing, laughing, shouting, jumping, talking strangely, acting weirdly, and even fighting did he realize that this was a mental hospital. There were eight wards for the accommodation of more than a thousand patients, and except for a few violent patients in a locked ward, most patients were free to move about the campus. There were only four physicians, five nurses, and a handful of attendants, all of whom were retirees from the military healthcare services.

During the following decades, many patients, under the work and industrial therapy programs of the hospital, actively contributed to the development of Yuli town, e.g., developing lands along riversides to grow rice, cleaning the streets, canals and police stations, and building a swimming pool for the town's senior high school. The hospital and the community also invited each other to join many entertainment, sport, and festival events. The interaction between patients and the community was friendly and mutually beneficial. In fact, patients did not feel rejected in the community even though they were "outsiders" twice over: soldiers of Kuomingtang from Mainland China and psychiatric patients. Yuli Veterans Hospital and its patients became part of the life of Yuli by engaging in community development projects and, thereby, winning the acceptance of the local people.

**Figure 1:**
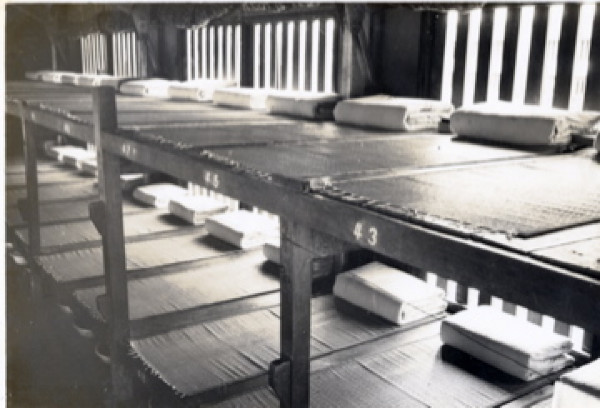
**The old inpatient ward using bunk beds (1960–1970s)**.

YVH occupies a curious place in the development of psychiatry in Taiwan. Since its establishment, the hospital has been neglected or "forgotten" because of its location, in the remote eastern Rift Valley, and its patients, mentally ill veterans under the jurisdiction of Veterans Affairs Commission, not the Ministry of Health (although the non-veteran inpatient population has been increasing since the 1980s). As a result, YVH is not only geographically peripheral, it is also peripheral to the psychiatry and public health mainstream of Taiwan. For example, an official report about the psychiatric services in Taiwan, 1980–1981, did not mention the 3,750 beds in YVH because, until 1986, it was under the jurisdiction of the Veterans Affairs Commission and not the Department of Health. Nor was the hospital included in "The Development of Public Health in Taiwan" [19], which was issued by the Ministry of Health in 1995. This omission was remarkable since Yuli had already been designated, since 1986, as the nuclear hospital in charge of mental health services for the eastern part of Taiwan [17,18]. YVH seemed to be neglected by Taiwanese psychiatry because it constituted a contrast to the mainstream goals of global psychiatry: deinstitutionalization and development of community psychiatry (Figure 1) [17,18]. It was not until 2000 that Taiwanese psychiatry began to recognize the change of YVH and its achievement in helping the institutionalized patients, who have long been regarded as difficult patients with no hope of recovery, to reintegrate into the community.

Today, YVH is one of seven nuclear mental hospitals that are responsible for mental health services within their respective catchment areas. YVH is unique, however, in that most of its patients come from all over Taiwan, which means that the task of community reintegration is particularly challenging.

A remarkable feature of Yuli illustrates a kind of reverse integration of psychiatric and general health services, with the gradual building of a large and modern general hospital alongside the psychiatric wards of the original hospital. The general hospital has all of the major clinical departments (Table 1) that one would expect to find in a general hospital, and provides extensive psychiatric and general health services (Table 2). Bed occupancy of the psychiatric departments is particularly high (Table 2).

**Table 1:** Clinical departments

Psychiatry
General Psychiatry
Geriatric Psychiatry
Psychosomatics
Rehabilitation Psychiatry
Community Psychiatry
Internal Medicine
General Medicine
Neurology
Gastrointestinal
Respiratory Medicine
Nephrology
Paediatrics
Surgery
General Surgery
Urology
Orthopaedics
ENT
Obstetrics and Gynaecology
Ophthalmology
Dentistry

**Table 2:** Facilities, resources and services

**Facilities (Beds)**	
Acute Psychiatric	120
Chronic Psychiatric	800
Acute general	95
ICU	6
Haemo-dialysis	26
Nursery	12
Nursing home	196
Residential Programs	446
Day care centers	200
Community Rehabilitation Centers	220

**Human Resources (Number)**	

Psychiatrists	31
Physicians	11
Surgeons	11
Dentists	1
Anaesthetists	2
Nurses	229
Psychologists	9
Occupational Therapists	19
Social Workers	12
Pharmacists	10
Dieticians	3
Radiology Technicians	2
Medical Technicians	7
Administrative Staff	31
Nursing attendants & other supporting staff	363

**Service Capacity (2006)**	

OPD	167,839/yr
ER	9,956/yr
Haemo-dialysis	15,236/yr
Acute Admissions	
General	3,6827/yr
Psychiatric	70/yr
ICU	178/yr
Surgical operations	1,335/yr
Obstetric deliveries	138/yr
	
Bed Occupancy	
Acute General	73.6%
Acute Psychiatric	95.5%
Chronic Psychiatric	97.2%

### Patient population

Three groups of patients are treated in YVH (Table 3). The first group consists of elderly veterans, most of whom were suffering from psychotic disorders, alcohol dependence, and depression when they were admitted during the first 25 years of the hospital. The majority of these patients are now more than 80 years old.

**Table 3:** Demographics of extended-stay patients of Yuli Veterans Hospital

	Veterans*	**Non-Veterans**	
	**Male**	**Male**	**Female**
Number of Patients (n)	605	1,058	767
Average Age (years)	71.1	45.8	51.1
Marital status			
Single	572	1,039	528
Married	30	13	71
Divorced	3	4	29
Widow/widower	0	2	45
Diagnosis			
Schizophrenia	92.0	91.3	97.4
Other	8.0	8.7	2.6

* All veterans are male

The second group is comprised of the mentally ill or mentally retarded children of veterans. For the first ten years after the Kuomingtang retreated to Taiwan in 1949, the military was preparing to launch a counterattack against the new Chinese Communist regime. Therefore, soldiers were not allowed to marry. When this prohibition was finally lifted the soldiers were comparatively old, as well as socially and economically disadvantaged because they were mainlanders and their pay was minimal. As a result, though they were free to choose their partners, many lower ranking soldiers could only afford to marry women who were marginal to Taiwanese society, e.g., mentally ill and mentally retarded women. Sadly, many of the children of these marriages also became ill. Currently, there are 34 mother-son and mother-daughter pairs and 39 pairs of siblings living in the hospital.

The third group of patients is different in terms of socio-economic and cultural backgrounds. This group is not made up of either veteran soldiers or their dependents. These patients have arrived at the hospital after drifting through the Taiwanese healthcare and welfare systems. YVH is rarely their first choice; on average they come to Yuli about 15 years after the onset of their illness. Many of them are seriously dysfunctional and treatment resistant. They have no family, or their families cannot afford to support them anymore. At the same time, their families still feel responsible, and do not want their ill relatives to become homeless. Thus, families, with the support of national health insurance or social welfare subsidies, send their ill relatives to YVH as a final option.

In Taiwan, the burden of long-term care for mentally ill persons falls upon the families, especially parents. When parents become elderly, it is almost impossible for their healthy children – who have their own families and careers – to take on the responsibility of caring for their ill siblings. Finally, the burden of care may crush parents, especially when there is no support from other family members or the wider community. However, when the mainstream of psychiatry is "deinstitutionalization" and "returning to the community" the question arose about where the community is for patients in YVH, and whether returning to it is a real possibility [17,18]. It was in this context that the concept of therapeutic community for patients was put it into practice beginning in 1990.

### The concepts of therapeutic community in the Yuli model

"Therapeutic Community" was a term used by Maxwell Jones as early as 1946 when he was working at Henderson Hospital, a facility for mentally ill veterans in England. Jones made the hospital a community by creating a democratic atmosphere, in contrast to the traditional authoritative style of hospital management [20]. In the Yuli model the concept of therapeutic community has been extended beyond the walls of the hospital and includes the town [21].

#### Hospital as therapeutic community

**Figure 2:**
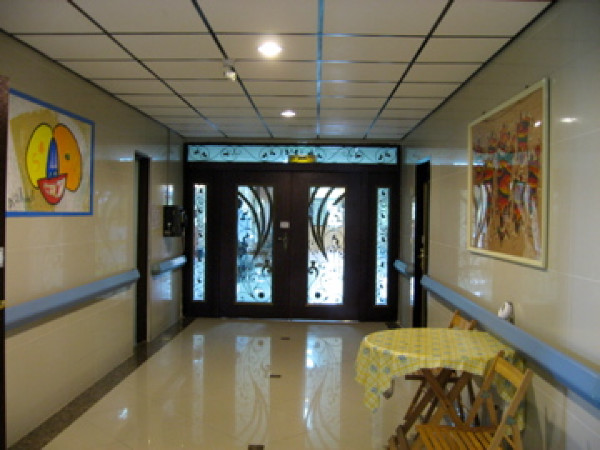
**The acute inpatient ward hallway**.

**Figure 3:**
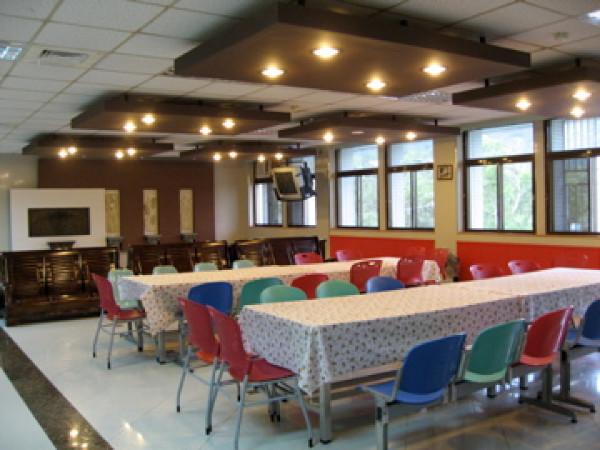
**The acute inpatient ward dining room**.

**Figure 4:**
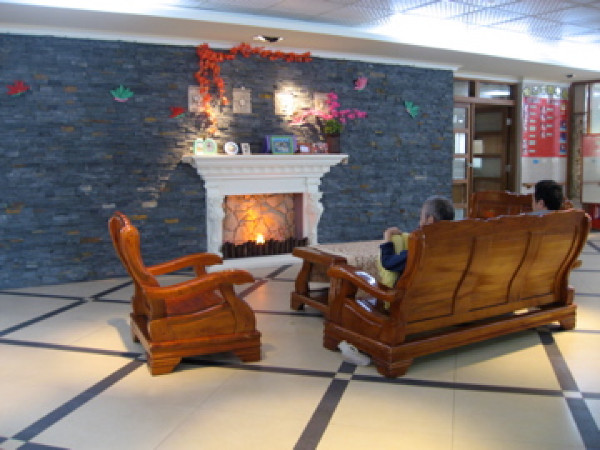
**The inpatient rehabilitation ward, lobby**.

**Figure 5:**
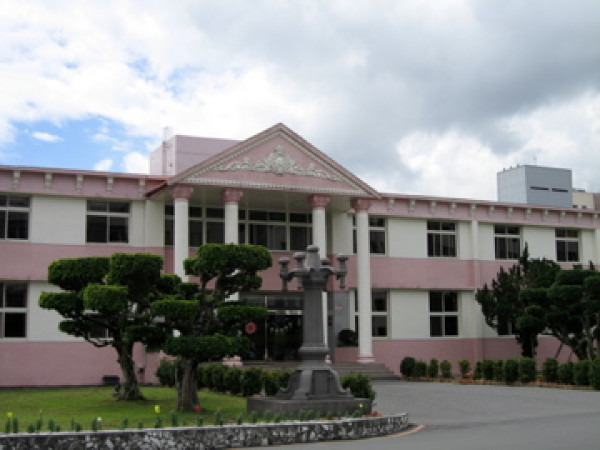
**Yuli Day Care Center**.

**Figure 6:**
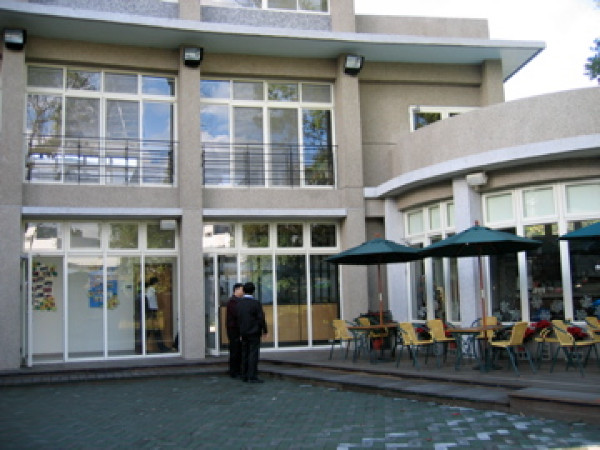
**Community rehabilitation center: Joint program with local church**.

Returning to the community is the ideal in the Yuli model. However, in practical terms, most patients cannot return to their community of origin. As a result, life in the hospital is made as similar as possible to life in the community. First, the one-size-fits-all custodial wards were diversified into units with different structures, as well as a continuum of treatment and rehabilitation modalities appropriate to patients' clinical reality. There are acute inpatient (Figures 2, 3), long-term rehabilitation (Figure 4) and day care units (Figure 5), community rehabilitation (Figure 6) and residential programs, and nursing homes. Patients may go through each of these stages during their stay in the hospital. Considerable resources have been put toward enriching the environment of the hospital campus, such as convenience stores, a post office, bakeries, a café, a recreational center, a restaurant, and a garden. Patients can move about the campus, interact freely with other patients and staff, and engage in therapeutic, recreational, or casual activities on the campus and in Yuli town. During these activities, they may develop close relationships with fellow patients and hospital workers. Together, patients and staff, have created a common life style and cultural identity in this hospital-community.

To help the most dysfunctional and treatment-resistant patients live in a more open and free space administrative support is crucial. At the management level it is necessary to have persistent efforts to achieve consensus concerning the goals and values of the hospital, and communicate that message to all levels of personnel. The goal of the hospital is not only to diversify and enrich the services it provides, but also to create a better future for patients, whether they continue to live in the hospital or are discharged to the community. The value of this hospital is that it offers patients a community in which it is possible to enjoy a meaningful life. This is what the reforms at Yuli Veterans Hospital have tried to achieve, even though families and society may continue to be pessimistic about the future of patients.

#### Yuli Town as therapeutic community

YVH is close to the center of life in Yuli town; it is only a 15-minute walk to downtown. After 50 years of interaction, a mutually dependent relationship has developed between the hospital and the town. Patients are seen as ordinary people as they mingle in the crowds, shop in stores, eat in restaurants or food booths, and join folk or religious activities. For example, patients – usually more than 100 at a time – may be seen in the Yuli traditional market, or in the Friday night market where they have frequent social exchanges with local inhabitants. Indeed, the inhabitants of Yuli have become accustomed to seeing patients participate in many aspects of community life, including working in family-run small businesses. Therefore, for patients to achieve social reintegration it is not necessary for them to return to their communities of origin. Rather, they have the opportunity to become part of Yuli by participating in the life of the community. Together, these processes build a sense of togetherness and feeling at home.

The most effective strategy to make the Yuli community open and supportive is through 'direct contact' by which the local inhabitants begin to know that patients are stable, safe, friendly, responsible, and competent. At the same time, patients have to be prepared to conform to the social norms and morals of Yuli. Familiarity with patients removes skepticism and fear. Therefore, as many opportunities as possible are created for patients to interact with the local inhabitants, including work therapy and supported employment programs which appear to be the best methods by which local inhabitants can gain in-depth understandings of patients. Thus, patients earn the respect, support, trust and acceptance of the inhabitants of Yuli.

### Components of the Yuli model

Yuli patients suffer from chronic illness and are severely dysfunctional and treatment-resistant. Rehabilitation activities are focused on efforts to address residual symptoms and dysfunctions, and reinforcing patients' strengths. Rehabilitation across time and activities is an integral part of daily life so that it can be beneficial. The four major components of rehabilitation in the Yuli model are: holistic medical support, vocational rehabilitation, case management, and the residential program.

#### Holistic psychiatric and medical services

Most patients in YVH suffer from schizophrenia, which is manifested in positive, negative and disorganized symptoms and, to a great degree, negative effects on neurocognitive, social and vocational functioning. As a consequence, intensive and continuous treatment and rehabilitation are offered. Psychiatrists work closely with colleagues such as case managers, psychiatric nurses, social workers, psychologists, occupational therapists, and non-professional mental health technicians who are trained to monitor and support patients on a daily basis. As patients live and work in the open campus and Yuli town they often face interpersonal stress and work pressures, which may influence their inner stability and result in relapse and crises such as instances of self-harm or aggression. Thus, a major challenge is keeping alert and sensitive in order to detect subtle changes of patients' emotions, behaviors, perceptions and thoughts. In addition to psychiatric services, patients can rely on various departments (e.g., general medicine, surgery, gynaecology, and dentistry) in the hospital, which is also the biggest general hospital in the area, to address their medical needs. Thus, continuous psychiatric and medical care is provided under one administrative roof, which minimizes problems with referral as well as the barriers associated with distance and transportation.

#### Case management

The main goals of case management are to mobilize all available resources to help patients remain clinically stable, get and keep jobs, and enjoy a satisfying life in the community. Therefore, assertive outreach, on-going, round-the-clock services are offered no matter where patients are (e.g., sheltered workplaces, community workplaces, markets, and the residential program houses) to ensure a continuity of care across time and functional domains (e.g., working, living, learning and leisure activities). Multidisciplinary teams, consisting of psychiatrists, nurses, social workers, clinical psychologists, occupational therapists, and mental health technicians in charge of vocational training and life coaching, are responsible for case management while also attending to a range of patients' needs concerning working, living, and learning in the community. The caseload is shared and is designed so that members of the team do not work alone, and there are supervisors and coordinators who help team members solve problems, boost morale, and secure on-going and consistent administrative support.

The development of interpersonal relationships and social connectedness are essential components of rehabilitation at Yuli. Ideally, every individual should be able to build up their own social network and mobilize the resources embedded in the network. However, mental illness, schizophrenia in particular, profoundly impairs patients' capacity to develop interpersonal relationships or assume responsible and productive social roles. Often it is not possible for patients to fit into or adapt to their surroundings, nor is it always possible to expect the community to accept patients unconditionally. In this vein, outreach assertive case management teams keep patients' clinically stable through offering continuous treatment and rehabilitation and also assist patients in creating social ties in their immediate environment. In fact, the case managers themselves are like bridging social ties for both patients and the community to help them understand and get accustomed to each other. Through outreach and case management, patients can gain support from the network and develop their own social connectedness gradually.

#### Vocational rehabilitation

**Figure 7:**
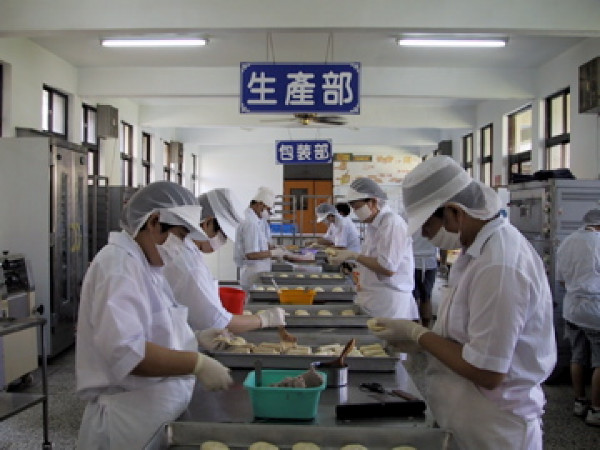
**Vocational rehabilitation: work training in hospital bakery**.

**Figure 8:**
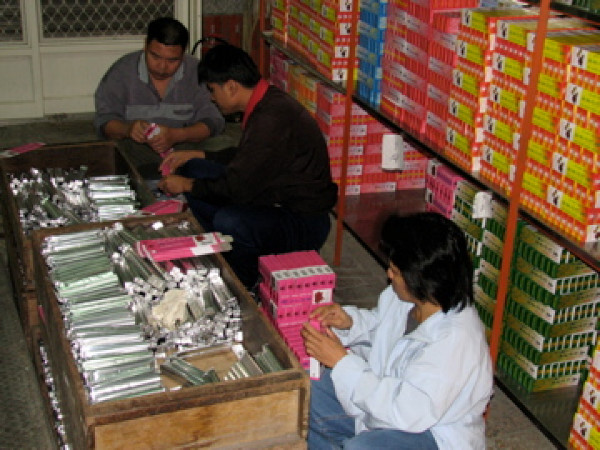
**Vocational rehabilitation: work training in community bakery**.

**Figure 9:**
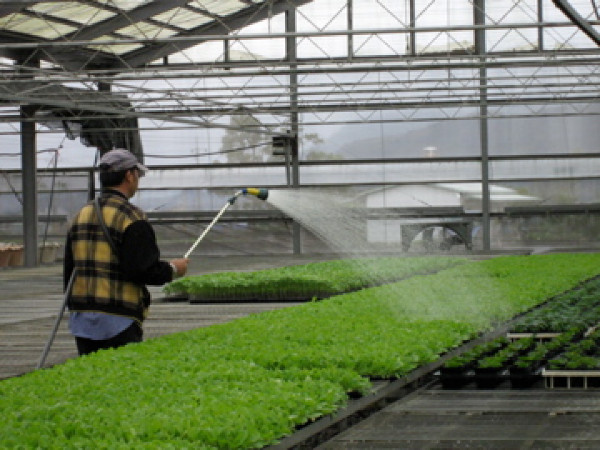
**Vocational rehabilitation: community employment in horticulture industry**.

Vocational rehabilitation aimed at gainful employment not only enhances patients' economic autonomy but also their place in the community and their dignity. Patients who are clinically stable, possess basic social and work skills, and are motivated to work – according to functional level – are assigned to one of three groups in the vocational rehabilitation program: hospital work training, community work training, and supported employment. Patients in the first two categories typically work part-time, entry-level jobs in hospital and community settings with minimal or subsidized pay. Currently, 213 patients are in hospital work training programs at 24 worksites, such as making bread in a sheltered bakery (Figure 7), doing daily chores at convenience stores, working in a horticulture garden, or washing clothes in a laundry. These work training activities combine the functions of traditional occupational therapy, prevocational training, and sheltered workshop. Community work training is intended for those patients who can work at least 20 hours per week, but whose productivity falls below the requirements of competitive employment. There are now 29 patients at 8 community work training sites, e.g., housekeeping at community hostels, doing daily chores at City Hall, and helping in a bakery (Figure 8). Patients in supported employment work at least 20 hours per week in community settings, and receive wages that are commensurate with those of a competitive job. At present, 52 patients work part-time at 25 sites, e.g., gas stations, supermarkets, restaurants, a hotel, and food stalls. Eleven patients work full-time (maximum of 48 hours per week) at 4 sites, e.g., doing daily chores at restaurants, bakeries, or in horticultural enterprises (Figure 9). In vocational rehabilitation patients are required to have good manners, dress appropriately, be well-groomed, and follow the advice and guidance of vocational counselors and employers. Vocational rehabilitation programs help patients internalize the structure and order of daily life, which, in turn, helps them to regain a sense of reality and control over their lives.

#### Residential program

##### Physical environment

**Figure 10:**
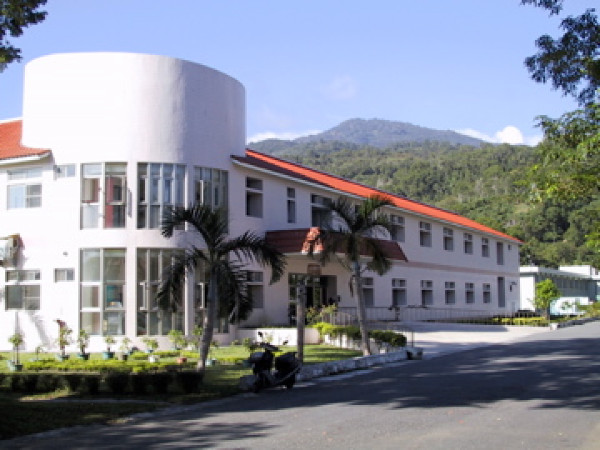
**The residential program**.

The residential program on the YVH campus is located in a pleasant and attractive two-storey building that has a spacious lobby, sunny rooms, a variety of recreational facilities, and an open garden (Figure 10). Each room can accommodate four persons, is furnished with four sets of closets and desks, and has a separate bathroom. In contrast to the inpatient wards, the residential facility has no locked doors or bolted windows. There are 203 patients in the program.

##### Rules and regulations

The residential program demands that patients take on more personal responsibilities. Unlike the inpatient wards, the program has relatively few rules about what residents must do on a daily basis and, aside from mental health technicians, there is no round-the-clock supervision by nursing staff. Residents are responsible for their personal hygiene, social behaviors, daily chores, money management, and participation in vocational rehabilitation programs. Residents take medication on their own, although supervision is available if needed. There is no regular roll call but residents have meals all together at designated time. In addition to working in the community, residents can spend their leisure hours in Yuli town on Friday nights and the weekends. Leave on weekdays is also permitted.

##### Social environment

Although patients enjoy more freedom, privacy, and access to the community, they are required to conform to rules that specify their rights and responsibilities in the residential program. It seems that patients, at least to some extent, continue to need external controls and structures in the process of reorganizing their lives which have been disrupted by mental illness. Residents have more opportunities to encounter fellow residents from different inpatient wards. And, they have more private space and more latitude for social contact than they have in the inpatient wards. At the very least, no verbal or physical aggression is allowed. Furthermore, the emphasis of staff-resident relationship is shifted from a custodian-patient relationship to a partnership. The staff focuses not only on monitoring changes of mental status, but also on providing guidance and empowering residents to participate in all the decisions that affect their daily lives. For example, residents have their own community council committee that meets regularly. They develop holiday festival programs or plan trips to Hualien or Taitung. They have also organized a self-help counseling team, which consists of senior residents with better functioning and providing emotional support and guidance for residents in terms of interpersonal relationships, how to negotiate with families, money management, and other personal issues.

In summary, the four components of the Yuli model help patients to recover two essential features of their lives: vocational life and ordinary daily routines. As this process of recovery evolves patients gradually regain inner stability, dignity, self-confidence, and a sense of control.

### Challenges faced by the Yuli model

A key challenge for the Yuli model arises from limitations in national health insurance funding of mental health services, which offers only nominal reimbursement for psychosocial interventions, nothing at all for vocational rehabilitation and case management, and questions the need for a long-term residential. The health authorities continue to see the residential program as merely a half-way house and not a stable and long-term housing service for those who have no other home. Owing to the slowdown of the Taiwanese economy and the resultant fiscal constraints, the government has reduced the budgets of public hospitals by 10–15 percent each year. This directly affects the pay of current hospital staff and impedes the recruitment and retention of new staff in this remote area of Taiwan. Tight financial management and identification of new sources of revenues will continue to be necessary in the immediate future.

In addition, there is a high turnover of staff, which is almost inevitable in this remote region of Taiwan. This increases the financial burden of the hospital (e.g. the need to pay higher salaries than hospitals in the Western counties) and also the workload of the experienced staff since they must train new staff all the time. The main challenge is to teach new staff the core values and mission of the hospital and how to work as part of a team. Continuous communication and coordination to reach consensus and ensure the continuity of quality services becomes an almost everyday task for the directors of different departments.

One of the major topics in training is how to manage crises when they occur, e.g., violence, self-injury, suicide, abrupt relapse, and leaving the hospital without permission. Since there is the advantage of geographical proximity between the hospital and the Yuli community, the mobile outreach team can reach most sites within 15 minutes to tackle the problem efficiently and avoid disturbing the peace of the neighborhood. The hospital has won the support and trust of the Yuli community by the timely and efficient management of crises. However, if an incident results in property damage or injury the administrative and legal systems must make enquiries. Staff face accusations from patients' families, administrative punishment, and legal suits. This reduces staff motivation to implement the program in open, non-secure community settings. Standard procedures of crisis management are now being formulated in an effort to persuade the Veterans Affairs Commission and the local legal system that if staff follow these procedures they should be protected from either administrative or legal consequences.

Since patients in YVH often have severe functional impairment and are often treatment-resistant it is necessary to provide intensive case management services to help patients move into the community and prevent crises from occurring. However, the limited quantity and quality of staff limits the capacity to help larger numbers of patients to live in the town, even when their level of functioning and clinical stability would suggest that they would be able to do so, with adequate support. The current goal is to have a further 100 patients living in Yuli in the coming two years, and to build a new residential facility in town. However, the biggest challenges are preparing patients and staff to deal with the possible resistance of the neighborhood, and building a safe and supportive environment for both patients and the neighborhood.

At the same time, the patients who are still living in the wards are not forgotten. In addition to improving their functional status and clinical stability through innovative rehabilitation programs there are plans to renovate the wards and other facilities to further improve their quality of life.

**Figure 11:**
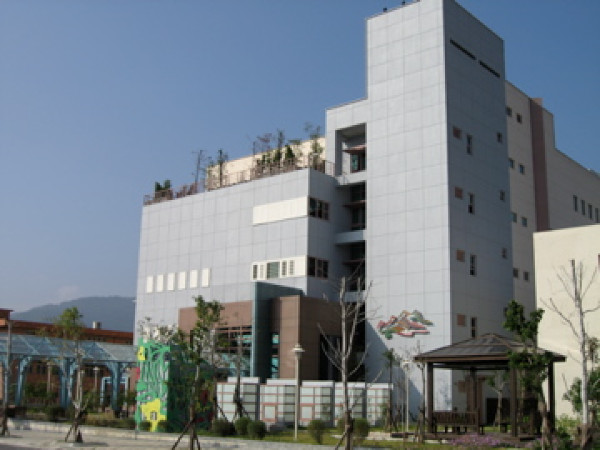
**Yuli Mental Health Research Center**.

To successfully lobby the government for more adequate funding for the programs, it is necessary to demonstrate the cost-effectiveness of this model in improving individual patients' quality of life, clinical stability and functioning. This will require the establishment of stable research teams that maintain an on-going focus on these issues. In Taiwan, research for community mental health service outcomes is still in an early stage. Developing this area of research will require collaborations among several disciplines, e.g., clinical psychiatry, epidemiology, anthropology, sociology, health economics, and health services research (Figure 11).

## Discussion

Why are the inhabitants of Yuli more willing to accept and support mentally ill persons than are other communities in Taiwan? What a local inhabitant said may answer this question:

"As a matter of fact, I had bad impressions about those patients who lived in Yuli Veterans Hospital when I was a child, because we often heard that patients were like time bombs and were dangerous and violent especially when they "escaped" from the hospital. But since I opened the supermarket I have been contacting them directly from time to time and found they are quite normal and friendly. And, some people even look lovely. They like to chat with you. Now I have heard very few negative things about these patients in Yuli. I think people here have already accepted them."

Interviews with Yuli residents revealed they have a common impression about patients, "neat, friendly, polite, and obedient to the norms". They said "You people help and supervise patients so that they can work and have activities outside hospital. It is good for patients and the community as well" [22].

The programs for people with severe and long-standing mental illness developed at YVH have sought to develop the concept of therapeutic community in a way that encompasses both the hospital and the community, being careful to increase patient autonomy as much as possible while maintaining sufficient structure to support enhanced functioning. Explicit efforts have been made to avoid the common problems reported elsewhere of communities complaining that hospitals have "dumped" patients into the community without adequate services and supports [23-25], and the common phenomenon "not in my backyard" [26,27]. Participation by YVH patients in the life of the Yuli community has been generally positively received, particularly by community members who have been in direct contact with patients. Among those community members with no or little direct contact with patients attitudes towards the mentally ill are not different from the general public in Taiwan, with attitudes shaped by biased and fragmented information about mental illness and the mentally ill [22]. Direct contact facilitates familiarity with the patients and enhances social support [28-31].

However, acceptance of and support for patients by Yuli residents is not unconditional even though YVH has been interacting with the local community for almost 50 years. For local people to accept patients living and working around them safety and mutual benefit must be assured. Local people may accept and even welcome the hospital, which can provide health care services and jobs, but having patients living and working in the community continues to be a challenge. For the past decade strategies have been devised to ensure that patients pose no threat to the peace of the community life and that they contribute to the growth of the local economy.

In the case of Yuli, the key element for the community integration of patients is the rebuilding of the structure and order of life of the patients [32] whose lives have been disrupted by severe and persistent mental illnesses [33]. The four components of the program are indispensable and interdependent parts of one service package, and must be operated simultaneously to benefit the patients to the greatest degree possible.

In the Yuli model, the holistic psychiatric and medical services mean that patients receive continuous, convenient and comprehensive medical and psychiatric care, enhancing psychological and physical health. The residential program provides decent housing and long-term and continuous support, supervision and life coaching from peer and service providers to maintain the structure and order of their daily ordinary life. Many patients who live with their families, in most case with their parents, become dependent, reluctant to go outside for social contact or work, and even have quarrels with their families and sometimes are violent towards them [34]. Also, those who live alone often become withdrawn from the outside world, receive no attention and support. They may live independently but actually have a detached life. Whatever the living circumstances of patients, case management, vocational rehabilitation, and psychiatric treatment face challenges and barriers. Patients need long-term stable residential services and also employment. If patients are unemployed or do not participate in vocational rehabilitation programs, the effects of psychiatric treatments, residential program and case management will have limited benefit. Vocational rehabilitation, including supported employment programs aimed at helping patients get and keep a job, is another key strategy. Work is a fundamental way to keep patients in contact with the real world and can help them regain self-esteem. The daily schedule and requirements of work strengthen the structure and order of their life [35]. Case management, in which the case managers serve as life coaches as well as bridging social ties between the patients and the community, is another pillar that upholds the structure and order of life of the patients. The case managers help patients follow the norms of community life and help the community inhabitants recognize that the patients are not only stable and friendly but also comprise an important part of the workforce in the local economy. Through case management patients have opportunities to build their social networks in peer groups, neighborhood, work sites, etc., identify themselves with the local community and its norms, and win the trust of local people. The four components work together to help patients settle themselves physically, psychologically, and socially in Yuli.

In the Yuli model, patients have access to medical and psychiatric services, case management, residential programs, vocational rehabilitation and supported employment programs under one administrative roof, and thus avoid the fragmented and disconnected services that are found elsewhere in Taiwan, where patients and their families get lost and frustrated in the maze of programs and systems, which usually only provide time-limited services and supports because of budget constraints. Inevitably, there are problems with transferring patients and service coordination and service efficiency and effectiveness are compromised.

One of the issues facing YVH is how to train the multidisciplinary staff to work together, particularly those who are accustomed to working independently in hospital settings. It is quite different to practice in the community settings for many professionals. Patients may behave very differently in the community than in the hospital. Therefore, it is necessary to rely on colleagues who observe patients across time, and in different places and contexts to put together all the pieces, and make certain that interventions are truly effective. The staff has to be empowered to work in the community. There should be a back-up plan to help first-line staff manage crises. Therefore, in addition to seminars and workshops, psychiatrists, nurses, occupational therapists, and other staff should be required to attend on-site trainings, for example, at patients' work sites, market, restaurants and anywhere the patients are. There also should be daily supervision and coordination to make the whole service work effectively. There are always conflicts of different viewpoints, opinions, approaches and personalities. Leaders usually spend a great deal of their time and effort to coordinate different sectors and help solve conflicts. Leadership training should be mandated. It requires long-term enduring efforts to search for and train the right persons as team leaders.

The experience of Yuli indicates that the structure and order of life are critical for patients to maintain internal control and thereby restore stable life and employment. This diminishes community fear and rejection and has contributed to the Yuli community's general acceptance of patients. Patients identify Yuli as their second home and the community accepts the patients as part of Yuli.

**Figure 12:**
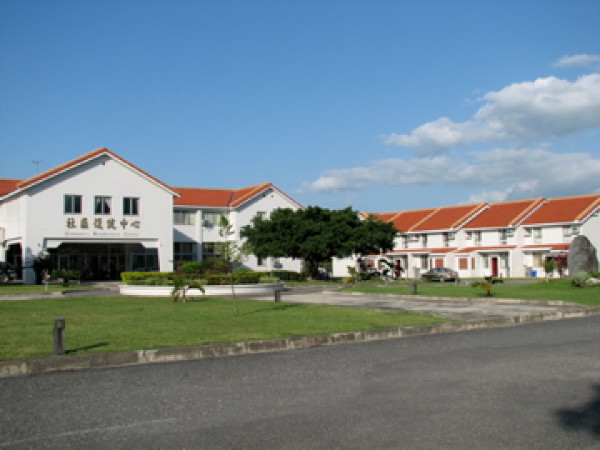
**Nursing homes for long-term care**.

For patients, most of whom are originally from other parts of Taiwan, the process of identifying Yuli as their new home is long and difficult. But as they become accustomed to life in Yuli, they come to think of themselves as inhabitants of Yuli. "I have stayed here (Figure 12) for more than 10 years. I returned those pressures and frustrations of my past life in Taipei, more and more, back to Taipei. To be honest, I already feel myself like a Yuli person, no more Taipei person, because I cannot any longer feel fit in the life of Taipei. Even the manner in which I walk has become quite different from Taipei people. I am used to walking around slowly here. You know. Even the angle of my legs and steps is so different from Taipei people.... After I moved here, at every sleepless night I feel the moon hanging up in the sky is shining solely for here" said one of the patients.

## Conclusion

The Yuli model demonstrates that there are multiple ways in which mental health system reform, and particularly the reform of mental hospitals, may be achieved. In many Asian countries that have traditionally relied on mental hospitals as the mainstay of often limited mental health services the call to close mental hospitals is neither realistic nor desirable. The Yuli model, which particularly focuses on the needs of people with long-standing illness and prolonged hospitals stay, illustrates one approach to linking hospital and community in a creative and constructive manner.

## Competing interests

The authors declare that they have no competing interests.

## Authors' contributions

C-Y Lin wrote the first draft. All authors contributed to the writing of subsequent drafts and have approved the final version.
